# Editorial: Trends post-COVID-19 attack: the cardiocerebral system safety remains of utmost concern

**DOI:** 10.3389/fphys.2023.1224550

**Published:** 2023-07-11

**Authors:** Moni Nader, George Haddad, Jacobo Elies, Sriharsha Kantamneni, Firas AlBadarin

**Affiliations:** ^1^ College of Medicine and Health Sciences, Khalifa University, Abu Dhabi, United Arab Emirates; ^2^ Center for Biotechnology, Khalifa University, Abu Dhabi, United Arab Emirates; ^3^ Howard University, Washington, DC, United States; ^4^ University of Bradford, Bradford, United Kingdom; ^5^ Cleveland Clinic Abu Dhabi, Abu Dhabi, United Arab Emirates

**Keywords:** COVID-19, cardiovascular, brain, long term sequelae, treatment

Survivors of COVID-19 infected individuals continue to suffer from a myriad of long-term sequelae that affect most organ systems of the body. To a higher degree, the preponderance of cardiovascular and cerebral sequalae of COVID-19 infection requires an in-depth analysis of these occurrences to decelerate or halt its expansion. Whereas cardiovascular manifestation of acute COVID-19 infection, including myocardial infarction, pericarditis, vascular thromboembolism, and cardiac arrhythmias, have been well described, (Abdel Moneim et al., 2022), longer-term consequences following recovery from symptomatic or asymptomatic infection remain incompletely understood. Given the large number of patients who survived COVID-19 infection worldwide, describing aberrations in cardiovascular and nervous system physiology may provide insight into mechanisms and potentially therapies for this illness.

This Research Topic of the Journal includes original studies and reviews that provide novel insight into the genesis of cardiac and neurological consequences of COVID-19. Such data may illuminate the development of novel therapeutic interventions to these patients.

In a review, Batta et al., addressed the cardiovascular symptoms in COVID-19 patients with a focus on vascular dysfunction, arrhythmias, myocardial ischemia, and discussed the most updated recommendations for the treatment of COVID-19. We previously reported the presence of almost all the receptors of SARS-CoV-2 on cardiomyocytes which makes the heart a favorable target for this virus (Abdi et al., 2022). Batta et al. indicated that the vascular endothelial dysfunction is involved in the pathogenesis of SARS-CoV-2 and hence the activation of pro-inflammatory cytokines leading to increased vascular permeability and thrombosis in many organs. Tachycardia was the most common cardiac presentation associated with SARS-CoV-2 infection, along with arrhythmias and conduction blocks, myocardial ischemia and injury, and hypertension. Interestingly, the authors reported that the elevated ACE-2 expression on endothelial cells of COVID-19 patients’ lungs indicates an elevated pro-hypertensive angiotensin II level leading to vasoconstriction and aldosterone-driven hypervolemia. Thus, the use of renin-angiotensin-aldosterone inhibitors in hypertension treatment of patients infected with SARS-CoV-2 was cautioned to avoid exacerbated cardiovascular clinical outcome.

An article from Gonzalez-Gonzalez et al. reviewed the application of Virchow’s Triad in detail for the risk of developing stroke and related intravascular thrombotic diseases in the context of COVID-19 infection. The authors discussed each part of Virchow’s triad in detail, such as hypercoagulable state, vascular damage, and intravascular stasis of blood. They looked into literature on the effects of COVID-19 infection for the formation of intravascular and intracardiac clots (leading to stroke), formation of cardiac sequelae and autopsy studies reporting elevated markers in ventricular myocardium. The authors reviewed the risk factor for stroke development, differences between ischemic vs. haemorrhagic stroke and frequent complications of COVID-19 patients such as pulmonary embolism. The authors also discussed the current treatment plans and recommended some differential treatment approaches for COVID-19 infection patients concerning known mechanisms of Virchow’s triad. Finally, the authors discussed the outcomes and long-term consequences of COVID-19 infection and the cardiovascular effects of COVID-19 vaccines.

The work from A. Mujalli and co-workers investigated genetic pathways in patients with severe COVID-19 and comorbidities, by means of genome-wide transcriptomic datasets publicly available within the first year of the pandemic. Differential gene expression (DGE), gene ontology (GO), pathway enrichment, functional similarity, phenotypic analysis and drug target identification studies were conducted using a cohort of 120 COVID-19 patients, 281 patients with chronic comorbidities (153 CVD, 64 atherosclerosis, 33 diabetes, and 31 obesity), and 252 patients with different infectious diseases (145 respiratory syncytial virus, 95 influenza, and 12 MERS). In total, 29 genes were identified to contributing to the clinical severity of COVID-19 infection in patients with comorbidities. Remarkably, identified genes were found to be involved in immune cell homeostasis during innate immunity, mostly in monocyte and macrophage function. In addition, results from drug target identification studies show a mismatch between the currently used drugs in COVID-19 therapy and predicted drugs against identified genes.

Furtheremore, in this Research Topic of the Journal, Chan et al. examined the association of COVID-19 with heart rate (HR) and blood pressure (BP) variability during exercise in a cohort of 18 patients with prior COVID-19 infection (equally split between symptomatic and asymptomatic), and a cohort of 9 controls who were never infected with COVID-19. Using a rigorous experimental design, the investigators measured HR and BP at regular intervals before, during, and after submaximal exercise, and quantified HR and BP variability on time and frequency domains. Baseline HR and BP were not significantly different between groups (symptomatic vs. asymptomatic vs. controls), nor were they different after completing a bout of submaximal exercise at a comparable workload. However, HR and BP variability was blunted only in individuals with prior symptomatic COVID-19 infection, but not in controls or those with a prior asymptomatic infection, suggesting an underlying degree of autonomic nervous system dysfunction in affected individuals.

The authors are to be lauded for their elegant and clinically relevant work, despite the obvious limitation of small sample size, since it provides much needed insight into COVID-19-induced abnormalities in cardiac physiology. The current findings provide a potential explanation for exercise intolerance, a frequently reported long-term symptom among survivors of COVID-19, since blunting of HR and BP variability are markers of impaired parasympathetic nervous system and poor cardiovascular health.

In conclusion, the COVID-19 pandemic affected millions around the globe before it started abating with the advent of the emergent vaccines that were approved for use on emergency basis. The WHO declared the end of the pandemic after 3 years of its surge. While millions succumbed to this deadly respiratory infection, survivors from this illness, particularity those who were severely sick, are reporting cardiac and nervous abnormalities. We hope that this series provides new perspectives on the manifestations of COVID-19 in the heart, the brain, and the vasculature with the hope to guide therapeutic interventions for patients suffering from long term sequelae of SARS-CoV-2 infection.
